# Antenatal administration of hepatitis B immunoglobulin and hepatitis B vaccine to prevent mother to child transmission in hepatitis B virus surface antigen positive pregnant women: A systematic review and meta-analysis

**DOI:** 10.1097/MD.0000000000019886

**Published:** 2020-04-17

**Authors:** Zhe Chen, Min Zeng, Dan Liu, Lin Wu, Lingli Zhang

**Affiliations:** a Department of Pharmacy; b Evidence-Based Pharmacy Center; c Key Laboratory of Birth Defects and Related Diseases of Women and Children (Sichuan University), Ministry of Education; d Department of Obstetrics and Gynecology, West China Second University Hospital, Sichuan University, Chengdu, China.

**Keywords:** hepatitis B virus, immunoprophylaxis, meta-analysis, mother to child transmission, systematic review

## Abstract

**Background:**

This study aims at evaluating the benefits and harms of hepatitis B immune globulin (HBIG) and hepatitis B vaccine (HBVac) in preventing mother to child transmission in HBV surface antigen (HBsAg) positive pregnant women during antenatal period.

**Methods:**

Seven electronic databases including PubMed, Embase, Cochrane Library, China National Knowledge Infrastructure (CNKI), WanFang Database, Chinese Biomedical Literature Database (CBM), VIP Database for Chinese Technical Periodicals (VIP), and 3 clinical trial registry platforms were searched from inception date to December 2017. Only randomized controlled trials (RCTs) were included in this study. The Cochrane risk of bias tool was applied to assessing the risk of bias. The outcomes were analyzed by Review Manager 5.3 software.

**Results:**

Sixteen RCTs involving 2440 HBsAg positive pregnant women were included in the meta-analysis. Compared with placebo group, HBIG and HBVac group had a significant decrease in the number of newborns who were HBsAg positive (relative risks [RR]: 0.2, 95% confidence interval [CI] [0.18, 0.40], *P* < .00001) and HBV-DNA positive (RR: 0.25, 95% CI [0.09, 0.71], *P* = .010), and had a significant increase in the number of anti-HBs positive newborns (RR: 3.95, 95% CI [3.11, 5.00], *P* < .00001). After 1-year follow up, the number of HBsAg positive newborns continued to decline (RR: 0.09, 95% CI [0.04, 0.20], *P* < .00001) and the number of anti-HBs positive newborns continued to increase in HBIG and HBVac group (RR: 1.30, 95% CI [1.22, 1.38], *P* < .00001). Compared with HBIG group, HBIG and HBVac group had no significant difference in the number of HBsAg positive newborns (RR: 1.68, 95% CI [0.66, 4.30], *P* = .28), and had a significant decrease in the number of HBsAg positive newborns (RR: 0.31, 95% CI [0.12, 0.84], *P* = .02). Additionally, only 1 study reported 2 swelling cases, 4 studies were reported no adverse events, and 11 studies were not report adverse reaction.

**Conclusions:**

HBIG and HBVac could be an effective alternative for HBsAg positive pregnant women to prevent mother to child transmission. However, due to the limitations of the study, the long-term efficacy and safety of HBIG and HBVac still need long-term and high-quality research to confirm.

## 1 Introduction

Chronic hepatitis B (CHB) is a major global health problem, resulting in substantial morbidity and 600,000 deaths per year.^[1]^ Worldwide, it is estimated that 2 billion people have evidence of past or present infection with hepatitis B virus (HBV) and around 650,000 people die of CHB each year from the CHB.^[1]^ As estimated 240 million of 2 billion individuals are chronic carriers of HBV surface antigen (HBsAg) all over the world.^[2]^ Chronic carrier status represents a status of increased risk for chronic hepatitis, liver cirrhosis, and hepatic carcinoma.^[3]^

Mother to child transmission (MTCT) is the main way of HBV transmission in many countries of the world, especially in China and South-East Asia, which may occur during gestation period, perinatal period, or after birth.^[2,4]^ When the mother is infected during the first trimester, 10% neonates may occur HBV MTCT.^[5]^ When the mother is infected during the third trimester, 60% to 90% neonates may occur acute infection.^[5]^ If acute infection is acquired in the last trimester, preterm birth rate may increase. Furthermore, >60% pregnant women acquiring acute hepatitis B infection will transmit HBV to their children.^[6]^

Therefore, post exposure prophylaxis is recommended for neonates from all HBsAg positive mothers, regardless of the HBeAg or HBeAb status.^[7]^ HBeAg indicates the infectiousness, the higher concentration of HBeAg and the higher degree of infectiousness.^[8]^ It has been shown hepatitis B immune globulin (HBIG) and hepatitis B vaccine (HBVac) is an effective treatment method for neonates at birth to prevent MTCT of HBV. Although administration of HBIG and HBVac in neonates has significantly reduced HBV carrier rates, however, approximately 1% to 9% of vertical transmissions of HBV were not eliminated by these interventions.^[4]^

Abou et al^[9]^ have showed antenatal period might be a main access point for the antenatal population to benefit from HBIG in limited resource settings. Eke et al^[10]^ have carried out a systematic review suggesting that HBIG might gain benefits when used for prevention of HBV MTCT and prevent neonates from developing HBV infection. Antenatal prevalence of HBsAg determines recommendation for pregnancy vaccination.^[11]^ Some studies have recommended that administration of HBIG and HBVac to mother might prevent intrauterine infection during pregnancy, although there were some controversies for its efficacy.^[10]^

Therefore, this study aims to evaluate the benefits and harms of HBIG and HBVac in preventing of HBV MTCT in HBsAg positive pregnant women. Although antiviral medications also have a role in the prevention of HBV MTCT, it is beyond the scope of this review.

## 2 Methods

### 2.1 Literature search

Our research comprises of 3 English electronic databases (PubMed, EMBase, and Cochrane Library) and 4 Chinese electronic databases (WanFang Database, Chinese Biomedical Literature Database, China National Knowledge Infrastructure, VIP Database for Chinese Technical Periodicals). Three clinical trial registry platforms were used to find additional studies, including ClinicalTrials.gov, the World Health Organization Clinical Trials Registry Platform and Cochrane Central Registry of Controlled Trials.

The search strategy was specific for each database and included a combination of the medical subject headings and free text terms for (“hepatitis B vaccine” or “HBVac” or “hepatitis B immune globulin” or “HBIG”) and (“vertical” or “mother-to-child transmission”). The deadline of all retrieval was December 2017.

### 2.2 Inclusion criteria

The following studies were included: types of studies: randomized controlled trial. Participants: pregnant women who were HBsAg positive or HBeAg positive or both. Intervention: HBIG and HBVac. Comparison: HBIG, HBVac, no intervention, placebo. Outcomes: primary outcomes were the number of HBsAg positive, anti-HBs positive, HBV-DNA positive in newborns, secondary outcomes were the number of HBsAg positive and anti-HBs positive in newborns after 1-year follow-up, and adverse reactions.

### 2.3 Exclusion criteria

The following studies were excluded: observational studies. Studies with incomplete or missing information. Suspected or documented infection, such as HCV, HIV. Not Chinese or English literature.

### 2.4 Data extraction

Data were extracted from all included studies. Extracted information included: study information (author, published time), method (study design, information of quality evaluation), intervention (sample size, medicine, dose), outcomes. Two independent reviewers screened all the titles and abstracts to determine potential eligible articles. They independently and blindly applied the eligibility criteria to perform the final selection. If they could not reach an agreement, the final decision would be made based on a third reviewer.

Two independent reviewers screened all the titles and abstracts to determine potential eligible articles. They independently and blindly applied the eligibility criteria to perform the final selection. If they could not reach an agreement, the final decision would be made based on a third reviewer.

### 2.5 Risk of bias assessment

We used Cochrane risk of bias tool for RCTs. Six domains of this tool included random sequence generation, allocation concealment, blinding of participants and personnel, blinding of outcome data, incomplete outcome data, and selective reporting. The judgment was marked as “high risk,” “unclear risk,” or “low risk.”

### 2.6 Data analysis

Meta-analysis was conducted with RevMan 5.3, cochrane centre. The data were pooled and expressed as relative risks (RR) with 95% confidence interval (CI). Assessment of heterogeneity was done by I-squared (*I*^2^) statistics. The data were considered homogeneous if *I*^2^* *≤* *50% and analyzed with fixed-effect model. Otherwise, heterogeneous data was analyzed with random-effect model. Statistical significance in this study was defined as *P *≤* *.05.

Meta-analysis was conducted with RevMan 5.3. The data were pooled and expressed as RR or mean difference (MD) with 95% CI. Assessment of heterogeneity was done by *I*^2^ statistics. The data were considered homogeneous if *I*^2^* *≤* *50% and analyzed with fixed-effect model. Otherwise, heterogeneous data were analyzed with random-effect model. Statistical significance in this study was defined as *P *≤* *.05.

### 2.7 Ethical statement

As all analyses were grounded on previous publications, ethical approval was not necessary.

## 3 Results

### 3.1 Characteristics of the included studies

**Figure 1:**
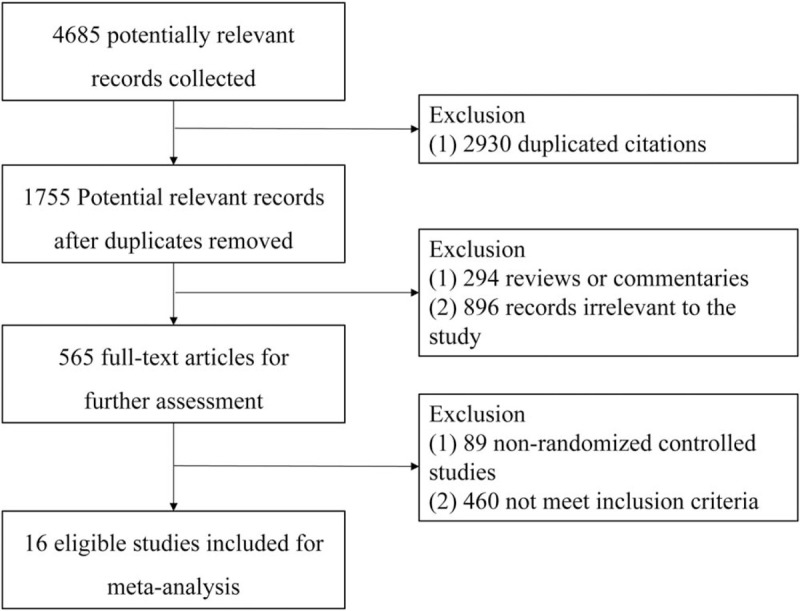
Flow diagram of selecting study.

A total of 4685 records were identified for initial screening and 16 eligible articles published between 2000 and 2017 were included in this meta-analysis (Fig. 1). All studies were reviewed by the ethics committee and signed informed consent. There was no significant difference in ages, sex, and disease course between 2 groups. Of these 16 studies, 15 studies were treated with 200 IU HBIG and 1 study was not reported HBIG dose at gestational. As for HBVac, 13 studies were treated with 10 mg HBVac at gestational, 2 studies were treated with 5 mg or 20 mg HBVac, respectively. And 1 study was not reported HBVac dose at gestational. At the same time, all the newborns were accepted adequate administration of HBIG within 12 hours of birth and a 3-dose succession of HBVac (Table 1).

**Table 1:**
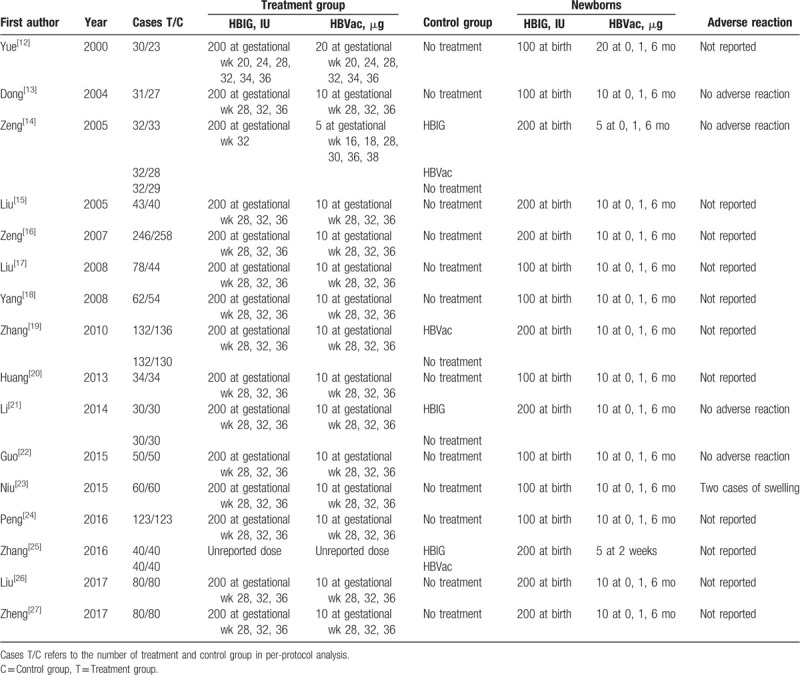
Characteristics of included studies.

### 3.2 Quality assessment

**Figure 2:**
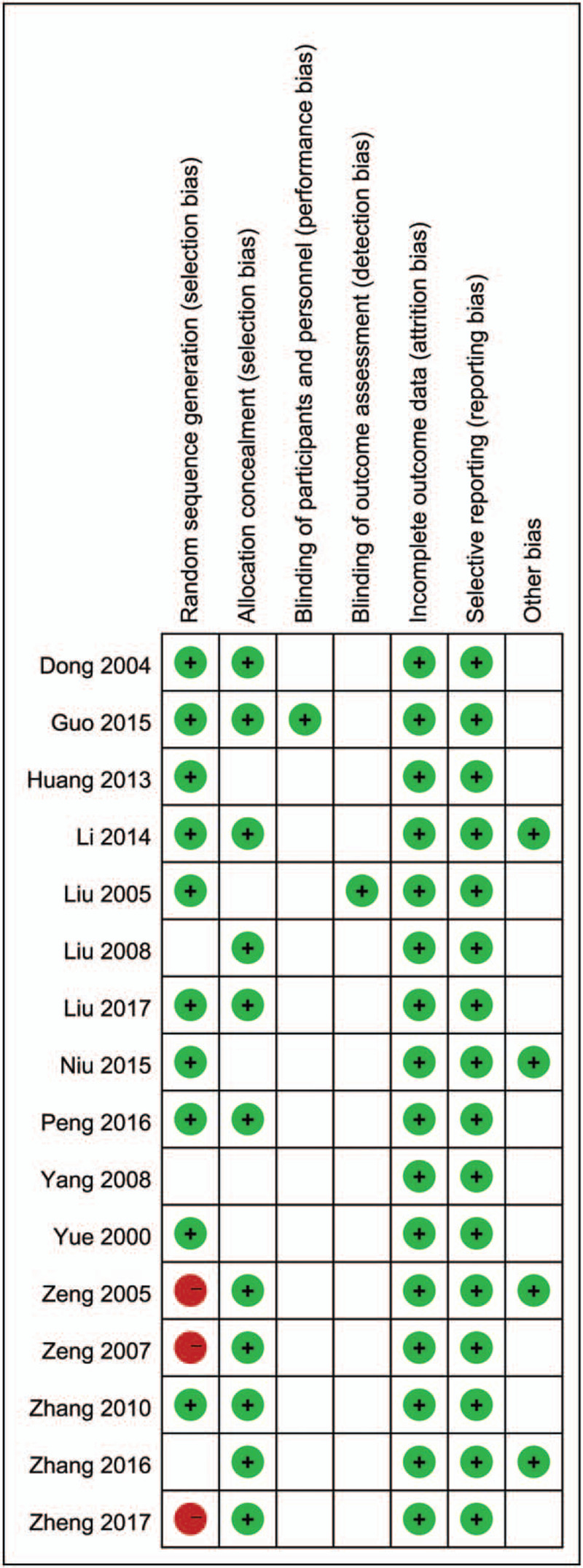
Methodological quality assessment.

According to Cochrane risk of bias estimation, 13 trials were mentioned random and 10 trials referred to the specific method of random. Eleven trials performed on allocation concealment. Only 1 trial performed blinding of participants and personnel assessment, as well as blinding of outcome assessment. All the trials reported on complete outcome data and selective reporting. Four trials were low risk in other bias (Fig. 2).

### 3.3 The number of HBsAg positive in newborns

**Figure 3:**
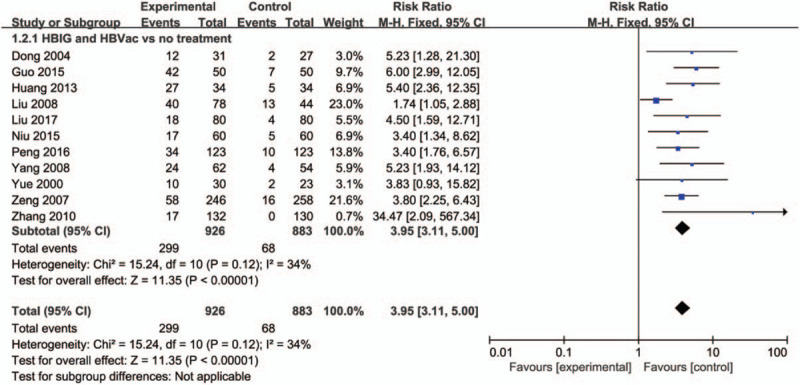
Forest plot of newborns positive for HBsAg. HBsAg = HBV surface antigen.

Among the 16 RCT studies, as for HBIG and HBVac versus no treatment, 15 studies contributed to this analysis. After pregnant women received the immunity prevention, there was a significant decrease in the number of HBsAg positive newborns (RR: 0.27, 95% CI [0.18, 0.40], *P* < .00001) with no significant heterogeneity (*P* = .02, *I*^2^ = 47%). As for HBIG and HBVac versus HBIG, 3 studies contributed to this analysis. After sensitivity analysis, there was no significant difference in decreasing the number of HBsAg positive newborns (RR: 1.69, 95% CI [0.66, 4.31], *P* = .27) with no heterogeneity (*P* = .91, *I*^2^ = 0%). As for HBIG and HBVac versus HBVac, 3 studies contributed to this analysis. Compared with HBVac group, HBIG and HBVac group had a significant decrease in the number of HBsAg positive newborns (RR: 0.34, 95% CI [0.13, 0.92], *P* = .03) with no heterogeneity (*P* = .63, *I*^2^ = 0%) (Fig. 3).

### 3.4 The number of anti-HBs positive in newborns

**Figure 4:**
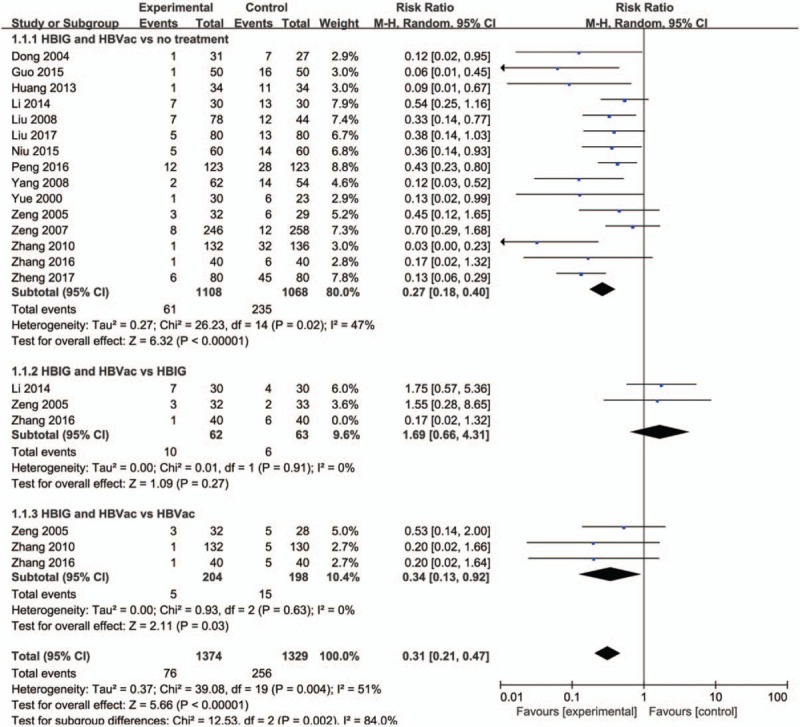
Forest plot of newborns positive for anti-HBs.

Among the 16 RCT studies, as for HBIG and HBVac versus no treatment, 11 studies contributed to this analysis. After pregnant women received the immunity prevention, there was a significant increase in the number of anti-HBs positive newborns (RR: 3.95, 95% CI [3.11, 5.00], *P* < .00001) with no significant heterogeneity (*P* = .12, *I*^2^ = 34%) (Fig. 4).

### 3.5 The number of HBV-DNA positive in newborns

**Figure 5:**
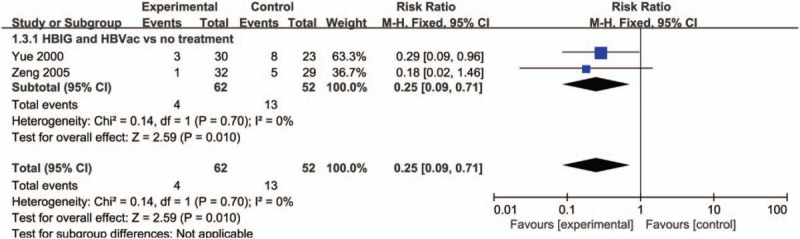
Forest plot of newborns positive for HBV-DNA. HBV = hepatitis B virus.

Among the 16 RCT studies, 2 studies contributed to this analysis between HBIG plus HBVac and no treatment. After pregnant women received the immunity prevention, there was a significant decrease in the number of HBV-DNA positive newborns. (RR: 0.25, 95% CI [0.09, 0.71], *P* = .010) with no significant heterogeneity (*P* = .70, *I*^2^ = 0%) (Fig. 5).

### 3.6 The number of HBsAg positive in newborns after 1-year follow-up

**Figure 6:**
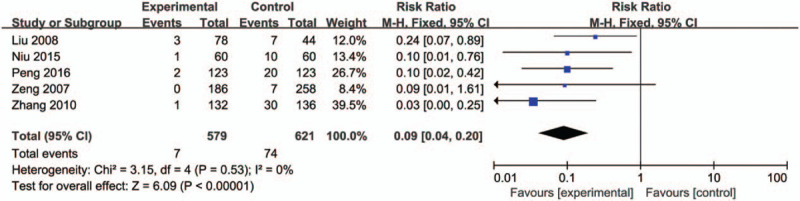
Forest plot of newborns positive for HBsAg. HBsAg = HBV surface antigen.

As for HBIG and HBVac versus no treatment, 5 studies conducted 1-year follow-up for newborns. Compared with no treatment group, HBIG and HBVac group had a significant decrease in the number of HBsAg positive in newborns (RR: 0.09, 95% CI [0.04, 0.20], *P* < .00001) with no significant heterogeneity (*P* = .53, *I*^2^ = 0%) (Fig. 6).

### 3.7 The number of anti-HBs positive in newborns after 1-year follow-up

**Figure 7:**
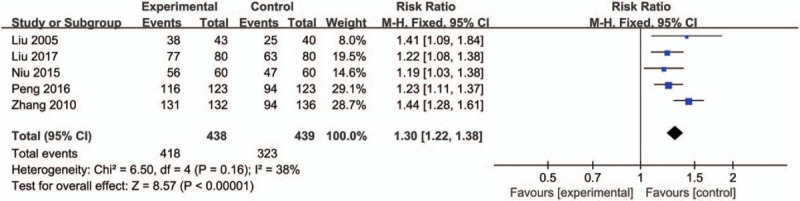
Forest plot of newborns positive for anti-HBs.

As for HBIG and HBVac versus no treatment, 5 studies conducted 1-year follow-up for newborns. Compared with no treatment group, HBIG and HBVac groups had a significant increase in the number of anti-HBs positive newborns (RR: 1.30, 95% CI [1.22, 1.38], *P* < .00001) with no significant heterogeneity (*P* = .16, *I*^2^ = 38%) (Fig. 7).

### 3.8 Adverse reactions

Among the 16 RCT studies, 11 studies did not report the adverse reaction. Only 1 study reported 2 swelling patients.^[23]^ Four studies reported no adverse events^[13,14,21,22]^ (Table 1).

## 4 Discussion

Overall, this systematic review included 16 randomized clinical trials involving 2440 subjects. About half of the trials (7 trials) included HBeAg-positive mothers.^[12,14,15,21,23,25,26]^ As for HBIG and HBVac versus no treatment, there was a significant decrease in the number of both HBsAg positive newborns (RR: 0.27, 95% CI [0.18, 0.40], *P* < .00001) and HBV-DNA positive newborns (RR: 0.25, 95% CI [0.09, 0.71], *P* = .010), and a significant increase in the number of anti-HBs positive newborns (RR: 3.95, 95% CI [3.11, 5.00], *P* < .00001) after birth. In terms of HBIG and HBVac versus HBIG, there was no significant difference in the number of HBsAg positive newborns (RR: 1.68, 95% CI [0.66, 4.30], *P* = .28) after birth. As for HBIG and HBVac versus HBVac, there was a significant decrease in the number of HBsAg positive newborns (RR: 0.31, 95% CI [0.12, 0.84], *P* = .02) after birth. After 1-year follow-up, the number of HBsAg positive newborns went on decreasing (RR: 0.09, 95% CI [0.04, 0.20], *P* < .00001) and the number of anti-HBs positive newborns went on increasing (RR: 1.30, 95% CI [1.22, 1.38], *P* < .00001). Additionally, no serious adverse reaction was reported. Therefore, HBIG and HBVac could be efficacy for pregnant women and newborns throughout administration and follow-up period.

Among the outcomes, as for HBIG and HBVac versus no treatment, the number of HBV-DNA positive in newborns was analyzed with only 2 studies and 114 neonates. So, this result needed to be further verified. Moreover, a few RCTs conducted a 1-year observation period and did not follow up the neonates in long-term. And the methodological quality of clinical trials needed to further improve.^[10]^

HBIG is widely used to confer passive prophylactic immunity against the HBV, replying on the ability of anti-HBs to eliminate hepatitis B virus.^[28]^ The possible mechanism is that HBsAb in HBIG can bind HBsAg and activate the complement system, then strengthening humoral immunity, reducing HBV levels.^[29]^ It not only can clear the circulating HBV and reduce the viral load in the maternal blood, but also can prevent and decrease HBV multiplication in the maternal body.^[30,31]^ After administration of HBIG, protective hepatitis B antibodies are transmitted to the fetus, which prevent intrauterine infection of the fetus by the HBV.^[30]^

We also recognized the limitations of this study. Firstly, only one trial performed on personnel assessment, blinding of outcome assessment, and participants. So we could not evaluate the risks of bias of the results. Furthermore, this study was not registered in a database. But before we started the study, we had made a predefined protocol. Thirdly, we searched all the electronic databases from inception date to December 2017. After screening the studies, we included 16 RCTs from 2000 to 2017. About 17 years from the first publication and last one may affect the results due to the time factor. Fourthly, because most of the trials did not report the adverse reaction, we could not confirm the safety for pregnant women and newborns. Fifth, we reviewed the 16 included RCTs, but we could not be sure that HBIG and HBVac were the same contents. Additionally, HBV infection is the major cause of end-stage liver diseases in China, among which 30% to 50% owes to MTCT and are associated with increased risk of morbidity and mortality later in life.^[32]^ Antiviral therapies could also provide benefits in HBsAg positive pregnant women, but this review was not designed to assess the efficacy of these agents. And we find no comparative benefits and harms between HBIG+HBVac and antivirals, so we could not draw comparative conclusions. Network meta-analyses could be used to compare the efficacy and safety between HBIG+HBVac and antivirals.

## 5 Conclusions

In summary, this systematic review and meta-analysis indicates that HBIG and HBVac could be an effective treatment for HBsAg positive pregnant women to prevent mother to child transmission. However, due to the limitations of the study, the long-term efficacy and safety still need to confirm by long-term and high-quality research.

## Acknowledgments

The authors thank Group of People with Highest Risk of Drug Exposure of International Network for the Rational Use of Drugs, China for providing support to coordinate circulation of the manuscript to all co-authors and collect comments from all co-authors.

## Author contributions

**Conceptualization:** Lingli Zhang.

**Data curation:** Zhe Chen, Min Zeng, Dan Liu.

**Methodology:** Min Zeng, Dan Liu, Lin Wu.

**Project administration:** Dan Liu, Lin Wu.

**Resources:** Lin Wu.

**Software:** Zhe Chen, Min Zeng.

**Visualization:** Lin Wu.

**Writing – original draft:** Zhe Chen.
